# A mixed methods protocol to evaluate the effect and cost-effectiveness of an Integrated electronic Diagnosis Approach (IeDA) for the management of childhood illnesses at primary health facilities in Burkina Faso

**DOI:** 10.1186/s13012-016-0476-5

**Published:** 2016-08-04

**Authors:** Karl Blanchet, James J. Lewis, Francisco Pozo-Martin, Arsene Satouro, Serge Somda, Patrick Ilboudo, Sophie Sarrassat, Simon Cousens

**Affiliations:** 1 Faculty of Public Health and Policy, London School of Hygiene & Tropical Medicine, London, UK; 2 Faculty of Epidemiology and Population Health, London School of Hygiene & Tropical Medicine, London, UK; 3 Centre Muraz, Bobo-Dioulasso, Burkina Faso

## Abstract

**Background:**

Burkina Faso introduced the Integrated Management of Childhood Illnesses (IMCI) strategy in 2003. However, an evaluation conducted in 2013 found that only 28 % of children were assessed for three danger signs as recommended by IMCI, and only 15 % of children were correctly classified. About 30 % of children were correctly prescribed with an antibiotic for suspected pneumonia or oral rehydration salts (ORS) for diarrhoea, and 40 % were correctly referred. Recent advances in information and communication technologies (ICT) and use of electronic clinical protocols hold the potential to transform healthcare delivery in low-income countries. However, no evidence is available on the effect of ICT on adherence to IMCI. This paper describes the research protocol of a mixed methods study that aims to measure the effect of the Integrated electronic Diagnosis Approach innovation (an electronic IMCI protocol provided to nurses) in two regions of Burkina Faso.

**Methods/design:**

The study combines a stepped-wedge trial, a realistic evaluation and an economic study in order to capture the effect of the innovation after its introduction on the level of adherence, cost and acceptability.

**Discussion:**

The main challenge is to interconnect the three substudies. In integrating outcome, process and cost data, we focus on three key questions: (i) How does the effectiveness and the cost of the intervention vary by type of health worker and type of health centre? (ii) What is the impact of changes in the content, coverage and quality of the IeDA intervention on adherence and cost-effectiveness? (iii) What mechanisms of change (including costs) might explain the relationship between the IeDA intervention and adherence?

**Trial registration:**

Clinicaltrials.gov, NCT02341469.

## Background

Despite a large reduction in under-5 child mortality (from 180 per 1000 live births in 1990 to 83 per 1000 live births in 2015), sub-Saharan Africa failed to reach the Millennium Development Goal 4 target of 60 deaths per 1000 live births [1]. In 1999, the World Health Organization (WHO) developed the Integrated Management of Childhood Illness (IMCI) strategy [2]. This strategy provides an algorithm to guide health workers through a systematic clinical assessment of sick children with the aim of improving the diagnostic classification and the treatment of these children [3–5] and hence reducing mortality [4, 6].

However, effective implementation of IMCI is often constrained by poor adherence to the guidelines [7–9]. Previous studies have reported that adherence to the guidelines decreases over time due to inadequate initial training, shortage of staff and insufficient supervision [10, 11]. Takada et al. [12] have noted that healthcare workers typically find the IMCI chart booklet burdensome and try to work from memory, resulting in a decrease in quality of care. In addition, healthcare workers may omit sections resulting in incomplete assessments [4, 13]. Chaudhary [10] demonstrated that the adherence of health workers improved with supervision. However, regular supervision of health workers after training is often lacking [8, 13], partly due to the lack of resources.

Burkina Faso introduced the IMCI strategy in 2003. However, an evaluation conducted in 2013 found that only 22 % of nurses working in primary care facilities had been trained in IMCI [14]. Only 28 % of children were assessed for three danger signs as recommended by IMCI, and only 15 % of children were correctly classified [14]. About 30 % of children were correctly prescribed with an antibiotic for suspected pneumonia or oral rehydration salts (ORS) for diarrhoea, and 40 % were correctly referred [14].

Recent advances in information and communication technologies (ICT) holds the potential to transform healthcare delivery in low-income countries [15–17]. However, no evidence is available on the effect of ICT on adherence to IMCI. Small-scale studies conducted in Tanzania found ICT to be well accepted by healthcare workers during IMCI consultations [7, 16]. However, the experience with using such technology on a large scale is limited.

In 2010, Terre des Hommes (TdH), a Swiss non-governmental organisation, together with the Ministry of Health (MoH), launched the Integrated electronic Diagnosis Approach (IeDA) intervention with the objective of improving adherence to IMCI guidelines in public primary health centres in two regions of Burkina Faso. In this paper, we present the design of a mixed methods evaluation of this intervention.

Burkina Faso is composed of 13 regions and 63 health districts. The public health system is characterised by a three-tier service structure: (i) at the first level are the districts with 1535 health centres (Centre de Santé et de Promotion Sociale (CSPS)) and the 104 district hospitals (Centre Médical avec Antenne Chirurgicale (CMA)), (ii) at the next level are the nine regional hospitals (Centre Hospitalier Régional (CHR)), and (iii) finally, the third level is comprised of the three national teaching hospitals (Centre Hospitalier Universitaire (CHU)) [18].

The IMCI approach was implemented only at the first level of the pyramid, i.e. in health centres. These facilities deliver a minimum package of services defined by the Ministry of Health comprising both preventive (e.g. vaccinations, antenatal care, health education, and promotion of proper nutrition, hygiene and safe water) and curative measures (e.g. treatment of common illnesses, minor surgery, supply of essential medicine, maternal and child consultations). CSPSs are governed by a management committee (Comite de gestion) composed of members of the community. The district health management team is in charge of supervising CSPSs and analysing routine data collected in them [19].

### The Electronic Register of Consultations and the IeDA intervention

The ‘electronic register of consultations’ or ‘Registre Electronique de Consultations’ (REC) in French was designed in 2010 by TdH [20]. The REC software, based on the CommCare software language, is installed on the open access CommCare platform developed by Dimagi [21]. The REC guides health workers through the IMCI algorithm. By doing so, it aims to improve adherence of nurses to the clinical protocol and to provide the local health district and the MoH with routine data on the management of childhood illnesses. The first versions of the REC were piloted in 2011 and 2012 in 52 primary health facilities located in two districts in the Nord region and perceived by 90 % of users (nurses) as being a supportive tool during consultations [22]. An additional pilot district, Yako, was added in the Boucle du Mouhoun region in 2014 and 2015. Following the pilot phase, the MoH requested TdH to expand the implementation of the REC to the remaining health districts of both regions.

In order to do so, TdH launched in 2014 the IeDA intervention, which includes the following five components, delivered at district and health centre levels:

- Development and implementation of the improved versions of the REC in all primary facilities of the two regions (district level).
- Provision, with the Ministry of Health, of a 7-day training course on IMCI guidelines including 2 days on the use of the REC to all primary health workers working in each district to be covered (district level).
- Development of a quality assurance mechanism through which each district and health centre is encouraged to find appropriate solutions in response to their local needs (district and health centre levels).
- Supervise every month every health centre benefiting from the intervention and support health district authorities in their annual supervision of health centres (health centre level).
- Development of a health information system based on the data collected through the REC and fed back to the district managers and heads of health centres (district and health centre levels).

## Methods/design

### The evaluation methods

**Fig. 1:**
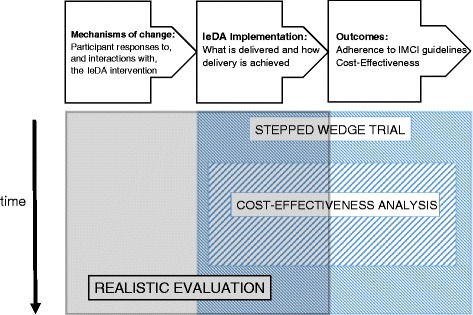
Evaluation mixed methods study framework

The IeDA intervention is being evaluated using a mixed methods study design composed of the following three interlinked studies (see Fig. 1):

1. A stepped-wedge trial to evaluate the effect of IeDA on the adherence to IMCI guidelines in primary health facilities
2. A cost-effectiveness analysis (CEA) to assess the value for money of the delivery of IeDA
3. A realistic evaluation to understand the implementation process, the mechanisms by which the IeDA intervention leads to change and to identify factors that may affect these mechanisms at the health centre and community levels

These three studies are taking place in a total of eight health districts across the Nord and Boucle du Mouhoun regions, with the three districts where TdH piloted the REC excluded from the evaluation.

### The stepped-wedge trial

**Fig. 2:**
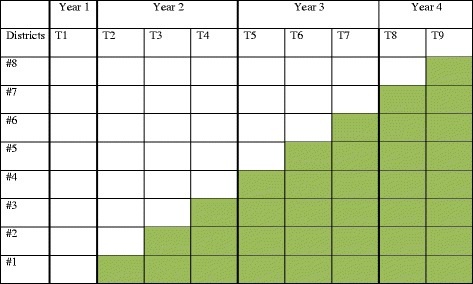
The sequence of districts receiving the intervention after each period of 4 months during the stepped-wedge trial

Stepped-wedge trials are a type of cluster randomised trial in which clusters receive the intervention at different time points and the order in which they receive the intervention is randomised [23, 24]. Some aspects of the intervention can only be delivered at the district level, and other aspects are most conveniently delivered by district and so, we are using a cluster randomised trial with districts as clusters. The two regions have 11 districts, three of which received the pilot intervention and so, the evaluation will be restricted to the eight remaining districts. The implementing agencies (the MoH and TdH) need to roll out the intervention in a phased implementation approach for logistical reasons, with the intervention introduced into one additional district every 4 months. Hence, we are using a stepped-wedge design, with the order in which the eight districts receive the intervention determined by computer-based, restricted randomisation (Fig. 2).

The intention was that the intervention would eventually be delivered to all primary health centres in all eight districts. Also, during the period preceding the intervention (i.e. in year 1 step 1), a first round of data collection would be conducted in the eight districts to provide pre-intervention measurements in all districts.

The aim of the trial is to determine whether the IeDA intervention increases adherence to the IMCI algorithm and hence improves classification, referral, prescription and counselling during under-5 child consultations in health centres. This will be determined by observation of child consultations followed by a repeat consultation by study staff in selected CSPS.

There are two co-primary outcomes, one task-based and one results-based, both measured at individual level using direct observation [25–27]:

1. Degree of adherence to IMCI guidelines, measured as a quantitative variable
2. Correct disease classification and prescription, measured as a binary variable

The secondary outcomes related to under-5 children consultations are as follows:

1. Correct identification of danger signs
2. Correct classification of children
3. Prescription of the correct medicine
4. Correct referral or hospitalisation
5. Correct counselling delivered to child carer

### Selection of primary health centres

The evaluation is being conducted in ten randomly chosen primary health centres per health district. The list of CSPSs selected for the evaluation has not been communicated to the implementing agency to reduce the likelihood that they target these health centres for more intensive support. Ten CSPS per district was chosen to give sufficient contextual variation, while minimising logistical difficulties and ensuring that some CSPS in each district were not selected to allow for selection concealment. Only primary health centres with staff trained in IMCI were considered for selection, and all hospitals were excluded. The intervention effect may vary by health centre workload, and so, the selection was stratified on the 2013 annual under-5 consultations caseload, as provided by the MoH. For the 337 CSPSs with staff trained in IMCI in the eight districts under evaluation, the annual caseload was transformed into a daily rate by dividing it by 261 days (i.e. the number of working days). Out of the 337 CSPSs, 13 were excluded, either because of missing caseload data (six CSPS) or because of a zero recorded caseload (seven CSPS). Out of the remaining 324 CSPSs, a further 54 CSPSs were excluded because they had caseloads of less than three cases per working day, which would have limited the number of consultations that could be observed in these facilities. Although these 54 CSPSs represented 17 % of the remaining 324 CSPSs, they only represent 4 % of the total caseload in 2013. In each district, five CSPSs were then randomly selected among those with fewer than seven cases per working day and five CSPSs were randomly selected among those with seven cases or more per working day (on average). Seven cases were chosen for the cutoff as this ensured there were at least nine CSPSs in each of the two strata in each district. Data will be collected in the same ten CSPSs per district during each step of the stepped-wedge trial during a 1- to 2-day visit (see the ‘Data collection’ section below).

#### Sample size calculation

Using the method described by Hussey and Hughes [28] and assuming that:

1. Ten health centres in eight districts will be visited every step.
2. A harmonic mean of ten children will present at each health facility over the course of a 1- to 2-day visit.
3. Sampling ten children per facility per round carries a design effect of 2.
4. The coefficient of variation between clusters is 0.3.

Then, there is 90 % power to detect an increase in the correct diagnosis and treatment from 25 to 33 %. If there is only a harmonic mean of four children at each health centre, there will be 98 % power to show an increase from 25 to 40 %.

#### Randomisation

There are eight districts (five in Boucle du Mouhoun region and three in the Nord region) in the stepped-wedge trial. Restricted randomisation was used to allocate one cluster (i.e. one district) to receive the intervention in each of the eight time periods [29]. The order in which the eight districts receive the intervention was subject to three constraints that address the following circumstances in which the trial was being performed:

1. An international organisation will be implementing a performance-based financing (PBF) intervention in four of the eight districts during the study, which may influence the performance of the health workers. In addition, two of the eight districts will act as ‘control’ clusters for their study, and so, it would be better if the IeDA intervention does not start in both these clusters before starting in any other cluster.
2. An international donor is prepared to provide support for two of the five districts in the Boucle du Mouhoun region, but they needed to start doing this soon.
3. There needs to be some balance in the randomisation order across the two regions.

Hence, the following restrictions were applied:

1. There must be one PBF intervention district and one PBF non-intervention district (either a control district or one excluded from the study) in each pair of randomised districts, i.e. in the first and second districts to receive the intervention, the third and fourth districts, the fifth and sixth districts and the seventh and eight districts. The difference in the average order of the PBF intervention districts and the non-intervention districts must be less than one. The first three districts must not include both PBF control districts.
2. At least one of the first two districts must be in the Boucle du Mouhoun region.
3. At least one of the first three districts and at least one of the last three districts must be from the Nord region.

Applying these criteria to the 40,320 possible randomisations resulted in 4224 acceptable allocations. For these 4224, no district was in a given position in the random order for less than 6.9 %, or more than 17.0 %, of acceptable allocations (292 and 720 acceptable allocations, respectively). If two districts were always next to each other in the order, then they should be treated as one district in the analysis, but among the 4224 acceptable allocations, the least and most common pairing of districts occurred in 6.8 and 42.8 % of acceptable allocations, respectively. We therefore judged that the restricted randomisation had not resulted in an overly constrained set of possible allocations and one of the 4224 acceptable allocations was chosen at random. This was done in Stata v13, by sorting the 4224 acceptable allocations on a variable containing random numbers generated by the ‘uniform()’ command and then choosing the allocation with the smallest random number; this was done by a statistical co-investigator (JL). This allocation will be communicated to the TdH and the MoH gradually, so they only know the next two districts to receive the intervention.

#### Data collection

As outcome measures, the trial is assessing the adherence to IMCI guidelines and the correctness of the illness classification, prescription, referral (if necessary) and counselling.

Each selected CSPS is visited for 1 or 2 days at each ‘step’ of the trial by a team of two staff from the evaluation team. One member of staff observes all under-5 children consultations performed by the local health workers while the other reassesses children after their consultation with the local health worker. Our teams notify the head of the health centre the afternoon of the day before our visit.

The first member of the evaluation team records information regarding each consultation in a detailed observation form. Three observation forms were designed, one per age category defined by the IMCI guidelines (i.e. 0–7 days, 7 days–2 months, 2 months–5 years) and pre-tested to include all questions or tasks that a healthcare worker should perform when following the IMCI guidelines. Each observation form is divided into sections corresponding to the different sections in the IMCI protocol for each age category. For instance, the observation form to observe consultations of children from 2 months to 5 years old includes sections for the assessment of danger signs: cough or fast/difficult breathing, diarrhoea, fever, ear problems, anaemia, nutritional status, HIV, immunisation and vitamin A uptake, referral, treatment and counselling. Observations are passive, and the observer never intervenes during the consultation. Information recorded in the observation form also includes whether the nurse referred to the IMCI chart booklet during the consultation or used the REC (if the district benefited from the intervention), as would be expected if IMCI was being implemented correctly. If the health centre has more than one nurse conducting child consultations, the observer follows the first child who is called for consultation.

Following observation of a consultation, the second member of the evaluation team, expert in IMCI, re-assesses the child independently using the REC to provide a ‘gold standard’ against which to compare local health workers’ classifications and treatment decisions. In case of discrepancies between the health worker and the IMCI expert, the final management decision is determined following discussions between the two of them.

In addition to observations and repeat assessments, a questionnaire, based on the WHO Service Availability and Readiness Assessment questionnaire [30], was developed to document the context in which the trial is conducted. This questionnaire is completed at each visit to a CSPS and captures information on the resources available for management of childhood illnesses according to the IMCI guidelines.

Two evaluation teams (each comprised of two members) were recruited for data collection. They had all previously been trained in IMCI by the Ministry of Health and had at least 5 years of experience of IMCI in their health centre. In addition, they underwent 2 weeks of training on the study methods, provided by the investigators.

#### Analysis

The stepped-wedge design can be analysed in different ways [31]. In the primary analysis, each step will be analysed using methods appropriate for a parallel cluster randomised trial, using district-level summaries of the outcome to calculate a difference measure. These difference measures will be combined across steps using inverse variance weighting. Hypothesis testing will be based on bootstrapping or a permutation test.

In the secondary analysis, the method described by Hussey and Hughes [28] will be used. As the outcome is likely to be relatively common, the risk difference will be modelled using generalised estimating equations at the level of the child consultation, with fixed effects for intervention versus control phase, time step and district and clustering at the level of the health centre.

Potential imbalances at the level of the child and the health centre between intervention and control phases will be investigated and any imbalances will be adjusted for. A priori, adjustment will also be made for district-level summaries of the outcome at baseline.

A detailed statistical analysis plan will be prepared before the end of data collection.

### Cost-effectiveness analysis

The aim of this sub-study is to estimate the value for money of implementing IeDA compared to current practice (implementing standard IMCI, based on the paper-based IMCI protocol) in the diagnosis of under-5 children in primary healthcare centres in Burkina Faso. The CEA, embedded within the IeDA stepped-wedge trial, will estimate both the cost per consultation and the incremental cost per child correctly managed associated with delivering IeDA compared to delivering standard IMCI. The perspective for the CEA is that of the healthcare provider, i.e. the MoH. For both standard IMCI and IeDA, costing will be undertaken using a micro-costing approach [32]. For each of the two interventions, the main components of the cost per consultation are as follows: (i) programme costs, i.e. costs associated with the implementation of the intervention that are incurred at the administrative level outside the point of delivery of healthcare, and (ii) patient costs or costs associated with the implementation of the intervention which are incurred at the point of delivery of healthcare [33]. To obtain an adequate estimate of the cost per consultation for each of the two interventions, programme costs will be allocated to patient costs using established cost allocation procedures [32].

The cost per consultation of IeDA and standard IMCI will be estimated using an observational before-and-after study design in the district of Ouahigouya, one of the districts included in the stepped-wedge trial. In this district, the costs per consultation of standard IMCI will be estimated during the 6-month period prior to IeDA implementation. The cost per consultation for IeDA will be estimated during the 6-month period after IeDA implementation.

For both arms, all resource consumption affecting programme costs at health centre and district level will be measured prospectively using surveys. This data will be supplemented with data from health centre and district accounts. All resource consumption affecting programme costs at national level will be measured retrospectively via interviews with agents who have participated in the implementation of both interventions (mostly MoH officers and, in the case of IeDA, also TdH coordinators). Resource consumption affecting patient costs will be measured using data recorded in the REC for every consultation in the ten Ouahigouya health centres selected for the stepped-wedge trial which is monitored in the period between 6 months before and 6 months after IeDA implementation. For each of the two interventions, the number of consultations monitored in the Ouahigouya health centres during this period is scheduled to be 80. Data collected in the REC for each patient consultation includes (i) duration of the consultation and (ii) medications prescribed in the consultation. An additional form will be added to the REC to measure consumables used during each patient consultation. To minimise bias in the measurement of resource consumption due to seasonal effects on healthcare delivery, data on resource consumption from all the consultations monitored in the stepped-wedge trial (whether standard IMCI or IeDA) 3 months before and 3 months after IeDA implementation in Ouahigouya will be used in the estimation of patient costs. Once resource consumption is measured, costs will be calculated using unit prices for each resource consumed. Depending on the specific type of resource, unit prices will be obtained from MoH databases, MoH price lists or supplier catalogues.

Regression analysis will be used to estimate the mean difference in costs per consultation between IeDA and standard IMCI. Cost-effectiveness modelling will be used to assess the incremental cost-effectiveness of IeDA versus standard IMCI.

### Realist evaluation

For Pawson and Tilley [34], issues of context and mechanism are crucial elements to consider in any realist evaluation as they help to explain ‘what works, for whom and in what circumstances’. For these authors, ‘what works’ is not of itself a helpful question as: ‘programs work (have successful “outcomes”) only insofar as they introduce the appropriate ideas and opportunities (“mechanisms”) to groups in the appropriate social and cultural conditions (“contexts”)’. Focusing on the realist dimensions enables us to examine the particular significance of ‘mechanism’ and ‘context’ more closely.

We will use longitudinal research, using a case study design [35], to enable us to understand the impact of key features or mechanisms for IeDA users and the context in which adherence to protocols is achieved (or not) over time.

In the case studies, the task of the research team will be to identify different context and mechanism configurations. These case studies will be purposively selected in relation to the performance of the health centre (in terms of adherence to the protocol) and the capacity of health centre staff to reflect on their experience. Informed by data from the stepped-wedge trial (high-performing and low-performing health centres), new cases identified from the list of health centres randomly selected in the trial will be added to our purposive sample. Four intervention health centres with high performance (positive cases) and four intervention health centres (deviant cases) with low performance will be selected.

Given the broad scope of the study and a desire to capture how IeDA works in situ, we will use a combination of methods:

1. Non-participant and participant observation of nursing and multi-disciplinary activities related to the use of IeDA (e.g. supervision from district managers, relation with pharmacist)
2. Post-observation interviews guided by issues arising from observations
3. Social network analysis to explore whether the nature of relationships between the actors of the health system will change [36, 37]
4. Key stakeholder interviews exploring views in general about the use, influences on use and impact of IeDA. We will record the interviews and code and analyse them using the software NVivo
5. Interviews with patients about their experience of IeDA
6. Survey questionnaire administered to REC users to understand their experience using the tool and perceptions on the benefits or constraints of REC
7. Review of relevant documentation (e.g. reports, statistics)
8. Field notes written during and after each visit

In the realist evaluation sub-study, the analysis will consist of identifying patterns and variables in one case study [38] through a ‘pattern matching’ process [39] and verifying the presence of the same pattern in other case studies where it was expected to obtain the same pattern, what Yin called ‘theoretical replication’ [38, p. 116]. Cross comparisons will also be made with deviant cases where other patterns are expected to be found for predicted reasons.

To enable comparisons, data analysed in the trial on the level of adherence, resource availability and health facility size and compared across areas and health centres will be used to identity well-performing heath centres and poorly performing health centres. This will allow qualitative cross-case analysis taking into account similarities and differences between health centres.

## Discussion

### Interconnected sub-studies

With the IeDA study, we aim for a holistic evaluation in which outcome, process and cost data are integrated in the analysis of the findings to maximise our capacity to understand the effect of the introduction of a complex intervention into a specific context.

In integrating outcome, process and cost data, we focus on three key questions: (i) How does the effectiveness and the cost of the intervention vary by type of health worker and type of health centre? (ii) What is the impact of changes in the content, coverage and quality of the IeDA intervention on adherence and cost-effectiveness? (iii) What mechanisms of change (including costs) might explain the relationship between the IeDA intervention and adherence?

To explore whether more intensive implementation of the IeDA intervention can generate better adherence outcomes, data collected on implementation parameters (e.g. fidelity and dose) will be used to categorise and rank health centres. We will calculate the correlation between outcome and the fidelity and dose of the IeDA intervention.

In addition, process and cost data will be integrated into the trial analysis to identify new hypotheses on factors influencing the performance of health centres. Two factors that may play an important role in performance are (1) the characteristics of health workers (e.g. their experience in IMCI, the time allocated to each consultation) and (2) the characteristics of health centres (e.g. team structure, leadership, resources available).

### Ethics

The Ethics Committee of the Ministry of Health of Burkina Faso and the Ethics Review Committee at the London School of Hygiene & Tropical Medicine have approved the trial. Risks and burdens to the patients and the nurse as a consequence of the study are minimal.

Every nurse at the health centre level who will be observed will be informed about the study and the procedures and written consent will be requested. Carers of children will give written informed consent to provide authorisation for one of the researchers to observe the consultation conducted by the nurse and for the other researcher to conduct a repeat consultation.

An information sheet in the local language will be read to all participants (carers who accompany children, nurses, MoH Officials, NGO and United Nations Officers). Participants will be encouraged to ask questions about the research. If consent is given, they will be asked to sign either their name or, if unable to do so, provide their verbal assent and print their thumb print, on the attached consent form.
